# Dietary intake and socio-economic predictors of inadequate energy and nutrient intake among women of childbearing age in Karamoja sub-region of Uganda

**DOI:** 10.1186/s41043-023-00351-z

**Published:** 2023-02-22

**Authors:** Christopher Muggaga, Ipolto Okello-Uma, Archileo Natigo Kaaya, David Taylor, Duncan Ongeng, Mugonola Basil

**Affiliations:** 1 Department of Food Science and Postharvest Technology, Faculty of Agriculture and Environment, Gulu University, P.O. Box 166, Gulu, Uganda; 2 Department of Food Technology and Nutrition, School of Food Science, Nutrition and Bio-Science Engineering, College of Agricultural and Environmental Sciences, Makerere University, P.O. Box 7062, Kampala, Uganda; 3 Department of Geography, Faculty of Arts and Social Sciences, National University of Singapore, Queenstown, Singapore; 4 Department of Rural Development and Agribusiness, Faculty of Agriculture and Environment, Gulu University, P.O. Box 166, Gulu, Uganda

**Keywords:** Dietary intake, Dietary recall, Food security, Karamoja, Maternal nutrition, Pastoralism, Uganda

## Abstract

**Background:**

Karamoja sub-region is the most food insecure part of Eastern Africa. The poor status of food security in the sub-region is reflected in the high rate of undernutrition among women of childbearing age (WCBA) and children under 5 years (CUFY). The sub-region is unique in Uganda in terms of cultural diversity, agro-ecology and rainfall pattern and agricultural practices. However, the influence of these unique characteristics on dietary intake of WCBA in the sub-region is less understood. Therefore, this study examined dietary intake and socio-economic predictors of inadequate energy and nutrient intake among WCBA in Karamoja sub-region.

**Methods:**

A longitudinal study design was used involving 755 WCBA in the harvesting season where 635 were followed-up in the planting season. Data were collected using 24-h recall, dietary diversity and socio-economic and demographic questionnaires.

**Results:**

Intake of energy and nutrients were generally significantly higher (*p* < 0.05) during the planting season than in the harvesting season. Irrespective of the district, physiological status and season, household consumption of plant-based foods was far higher than intake of animal-based foods dominated by starchy stables (76–100%), dark green leafy vegetables (70–100%) and legumes, nuts and seeds (22–97%) depending on the district. Majority of the respondents had two meals (breakfast: 65–100%; supper: 90–100%) with up to 45% of WCBA who consumed alcohol across meal times. .Overall on average, 57.7, 66.6, 78.5, 60.3, 67.7 and 93.7% of WCBA did not meet the recommended daily allowance (RDA) for energy, protein, calcium, iron, zinc and folic acid, respectively. Binary logistic regression revealed that inadequate intake of energy and nutrients significantly increased (*p* < 0.05) with the status of being lactating/breastfeeding and was influenced by age of WCBA, number of women married, education level and occupation of the household head depending on season.

**Conclusions:**

This study demonstrated that dietary intake of WCBA in Karamoja sub-region was inadequate. Age of WCBA, number of women married, education level and occupation of the household head and spouse and being a lactating/breastfeeding mother were the key socio-economic and demographic factors that influenced inadequate intake of energy and nutrients among WCBA.

## Background

Maternal undernutrition is endemic in most countries in sub-Saharan Africa (SSA), with more than 20% of women in the subcontinent having a body mass index of less than 18·5 kg/m^2^ [1]. The prevalence of maternal malnutrition is still high in SSA where maternal underweight exceeds 20% in some countries with exception of few countries such as Benin, Cameroon, Ghana, Lesotho, Rwanda, Swaziland and Togo [2]. Maternal undernutrition is responsible for several global challenges in which poor child survival, incidence of acute and chronic diseases, unhealthy development and low economic productivity [3], low birthweight, stunting and poor cognitive development [4] are implicated. Maternal malnutrition is of particular importance because of the fact that nutrition in the first 1000 days of human life is critical for subsequent development [5–8]. During this period, there is rapid growth and development, fastest rate of neurodevelopment of cognitive functions, maturation of all organ systems and establishment of metabolic patterns [8–11]. As such, maternal nutrition before and after conception is a major focus for government and development agencies, with national commitments in low-income and middle-income countries (LMICs), increased donor funding and civil society and private sector engagement [12]. Examples of such engagements include scaling up nutrition (SUN) as a framework for action 2011 [13], Multi-Sectoral Nutrition Strategy 2014–2025 [7], Global Governance for Nutrition and the Role of UNSCN [14] and Civil society participation in global public private partnerships for health [15].

Malnutrition is also a major development concern in Uganda, affecting all regions of the country. For instance, the previous three Demographic and Health Surveys (DHS) (2006, 2011, 2016) indicate that undernutrition is on decline but the progress is very slow. The report indicates that among women the prevalence of anaemia reduced from 49%in 2006 to 32%% in 2016 [16, 17]. It is generally recognized that women and children in Uganda continue to experience deficiencies of vitamin A and zinc [18] there is lack of national data on the extent of deficiency.

Karamoja sub-region in Uganda has disproportionately high prevalence of malnutrition driven by high levels of food insecurity. Regarding maternal nutrition, undernutrition among WCBA is disproportionately high while health indicators are poor. For instance, 32% of WCBA in the sub-region were found to be anaemic [17]. On the other hand, 30% and 12% of the population experience stress and crisis level food insecurity [19]. Food insecurity in the sub-region exists amidst very complex situations comprising marked differences in terms of ethnic groups [20–22], agroecological zones [21, 22] and livelihood strategies [23]. Added to this is seasonal availability of food [24, 25], which further exacerbates food insecurity and access to a well-balanced diet. The current study is quite unique because it was conducted in an area that has only one rainy season [26] that coincides with the planting season. Seasonal variation in food availability has far reaching implications for maternal nutrition and health. For instance, dietary diversity score (DDS) and nutrition status of women [27–29] as well as frequency of food consumption [30] by women were reported to be influenced by season. Coupled with this, season also affects nutrient intake levels [28, 30–32]. However, a critical analysis of previous studies indicates disparities in the extent to which seasonal variations affect various nutrition indicators, suggesting that other factors including geographical location, socio-economic, socio-cultural and demographic are important modifiers. The existing disparities in food and nutrition security situation in Karamoja sub-region [33] could partly be attributed to aforementioned complex situations, influencing nutrient intake among WCBA. However, empirical data to support dietary intake are largely lacking. Hitherto, it is widely acknowledged that nutrition of mothers has a great impact on their health, including the health of children [11, 34–38]. Accordingly, the current study focused on understanding the dietary intake and socio-economic predictors of inadequate energy and nutrient intake by WCBA. This study makes contribution to the efforts in improving maternal nutrition in communities that are resource constrained and have poor state of human development indicators such as Karamoja sub-region. Understanding dietary intake of WCBA such circumstances would constitute the basis for designing strategies to reduce the adverse consequences of food and nutrition insecurity on maternal nutrition.

## Methods

The main aim of the study was to investigate dietary intake and socio-economic predictors of inadequate energy and nutrient intake among WCBA in Karamoja sub-region. A longitudinal design was used. Accordingly, 755 WCBA participated during the first visit (October to December 2014) of the study corresponding to the harvesting season. Of these women, 635 were followed (April to June 2015) in a period coinciding with the planting season [39]. The sample size was established using a standard formula [40]. A multi-stage sampling technique was used to select study participants based on livelihood zones [22, 41, 42], geographical locations [23] and ethnic group inclusiveness [20–22]. Hence, Abim, Amudat, Kaabong and Moroto were selected in the first stage. In the second stage, simple random sampling was used to obtain two sub-counties per district. The selected sub-counties were Alerek, Morulem, Kathile, Sidok, Nadunget, Rupa, Loro and Amudat. In the third stage, two parishes were randomly selected from the sub-counties. In the fourth stage, two villages were randomly selected from each of the selected parishes. A total of 32 villages were selected.

Finally, assisted by members of village health teams and local council officials, participants in each physiological category (pregnant women, lactating mothers and non-pregnant women) in the selected villages were enrolled.

Data were collected with the help of trained research assistants who were resident in the selected districts and fluent in their respective local languages. These research assistants were able to interpreting the questions from English to the local languages during face-to face interviews. Dietary assessment was carried out using the Household Dietary Diversity Score-HDDS [43] and 24-hour dietary recall [44] to determine dietary quality and nutrient intake, respectively. Information on HDDS was obtained through a recall of all foods/drinks consumed in the household in the previous 24 h. Prior to conducting 24-hour dietary recall, the common staples: sorghum, maize, millet were also prepared by one of the households in different forms such as bread, boiled grains that are commonly consumed in the area. Similarly, sauces from green vegetables, beans were prepared. During the 24-hour recall interviews, the portion sizes of the solid foods consumed in the previous 24-h period were weighed (in grams). For all foods, which were liquid or semisolid such as porridge, tea and milk, water was poured in a calibrated cup to estimate the volume consumed. These were followed by calculations of energy and nutrient composition (carbohydrates, proteins, calcium, iron, zinc, folic acid) from the portion sizes of individual foods and ingredients consumed by each participant using the HarvestPlus food composition table for central and eastern Uganda [45]. To assess the socio-economic and demographic status of the women and their household, information on age, marital status, family type, occupation and education level were gathered.

Data on socio-economic and demographic characteristics and dietary diversity were analysed using descriptive statistics and expressed as the proportions. The mean energy and nutrient intake between seasons for each district were compared using paired sample t-test at 5% significance level (*p* < 0.05).

Differences in nutrient intake among districts and/or livelihood zones were determined by one-way analysis of variance (ANOVA) at 5% level of significance followed by separating the means using LSD method.

Data from the 24-hour dietary recall were first used to generate meal patterns of respondents and presented as line graphs. To determine the level of inadequacy of energy and nutrient intake, recommended daily allowances (RDAs) for energy [46] and nutrients [47] for each physiological status were used where “1” was assigned to respondents with adequate intake and “0” to respondents with inadequate intake. These data were summarized as proportion of WCBA with inadequate intake of energy and nutrients. Finally, a linear regression was used to determine socio-economic and demographic predictors of inadequate energy and nutrient intakes among WCBA. The regression model used is presented in Eq. (1):

1 $$Yi = \beta 0 + \beta 1X1 + \beta 2X2 + \ldots \beta nXn + \mu$$

where *Y*_*i*_ is the dependent variable describing the status of intake of energy, proteins, calcium, iron, zinc, folate and vitamin A. *β*_0_ is the regression constant; *β* is the regression coefficient; and *μ* is the error term. *X* denotes independent variables that were used to run the binary logistic regression. Before actual regression analysis, correlation analysis (Pearson) was performed to eliminate highly correlated dependent variables with coefficient greater than 0.70 [48]. Following correlation analysis, the following variables were used in the regression test: physiological status, age, marital status, family type, number of women married, occupation of the woman, occupation of the household head, education status of woman and education status of the household head. These variables, their description/measurement and the priori expected sign are presented in Table 1. Lastly, cut-off of < 23.0 cm to define undernutrition [49] was used to interpret variations in nutrition status of the WCBA during harvesting and planting seasons. Spearmans correlation was carried out to establish correlation between MUAC and nutrient intake.

**Table 1:** Variables, description/measurement and the priori expected sign

Dependent variable	Description	Variable coding/definition	Expected priori sign
*X*_1_	Pregnant	Pregnant women = 1, none pregnant women and lactating mothers = 0	+ or −
*X*_2_	Lactating	Lactating women = 1, none pregnant women and Pregnant women = 0	+ or −
*X*_3_	Age in completed years		+ or −
*X*_4_	Polygamous	Polygamous = 1, Monogamous = 0	+ or −
*X*_5_	Marital status	Married = 1, not married, widowed, single or divorced = 0	+ or −
*X*_6_	Occupation of the household head	Formally employed = 1, not formally employed = 0	+ or −
*X*_7_	Occupation of woman in the household	Formally employed = 1, not formally employed = 0	+ or −
*X*_8_	Education status of household head	Formally educated = 1, not educated = 0	+ or −
*X*_9_	Education status of the woman in the household	Formally educated = 1, not educated = 0	+ or −
*X*_10_	Number of women married	One woman = 1, more than one women = 0	+ or −

All the statistical analyses were performed using GenStat version 12 and IBM Statistical package for Social Scientists (SPSS) version 20 and STATA.

## Results

Socio-economic and demographic characteristics of the study participants are summarized in Table 2. Irrespective of season, majority of the participants were in the age category of 20–29 years (41–51%), followed by those in the age bracket of 30–39 years (25–34%). Abim and Amudat districts had the highest proportion of teenage mothers. Most of the WCBA surveyed were married accounting for 85.5–95%. More than half of WCBA were in monogamous relationships, except in Abim district where the proportion was much lower (12.5%) with about 60% in polygamous relationships (approximately double that in other districts). In all districts, subsistence farming was the major occupation employing 62–95% of WCBA. These women also commonly participated in tree cutting (for firewood and charcoal production) and brewing in Moroto and Kaabong. Generally, education attainment by WCBA was very low in Amudat and Moroto. More than 90% of WCBA in Moroto and Amudat, and 62.8% in Kaabong had not received any formal education. The proportion of those who had received formal education was highest in Abim, where more than 70% of participants had spent some time at school.

**Table 2:** Socio-economic and demographic characteristics of the respondents (%) segregated by district

Category	District			
	Abim	Kaabong	Moroto	Amudat
*Physiological status*				
Pregnant	33.9(12.2)	33.2 (8.5)	33.5 (11.5)	33.5 (14.2)
Lactating	33.9 (57.9)	33.7 (55.8)	36.8 (63.0)	37.8 (63.8)
Non-pregnant	32.3 (29.9)	33.2 (32.7)	29.7 (8.5)	28.6 (22.0)
*N*	192 (164)	190 (165)	185 (165)	185 (141)
*Age (years)*				
15–17	1.6 (0.6)	3.2 (3.6)	6.0 (5.7)	5.6 (3.6)
18–19	12.0 (8.6)	5.9 (6.7)	4.9 (7.6)	6.7 (6.5)
20–29	49.5 (51.5)	51.3(40.6)	51.6 (48.4)	49.4 (50.7)
30–39	33.9 (30.1)	33.7 (33.9)	24.7 (29.9)	30.9 (31.2)
40–49	3.1 (9.2)	5.9 (15.2)	12.6 (8.3)	7.3 (8.0)
*N*	192 (163)	187 (165)	182 (157)	178 (138)
*Marital status*				
Married	92.7 (93.9)	86.8 (84.8)	85.4(88.5)	95.0 (97.2)
Single	1.6 (1.2)	12.6 (12.1)	11.4 (10.3)	5.0 (1.4)
Widowed	4.1 (3.1)	0.5 (3.0)	3.2 (1.2)	(1.4)
Divorced	1.6 (1.8)			
*N*	192 (163)	190 (165)	185 (165)	180 (141)
*Family type*				
Polygamous family	60.6 (60.1)	35.3 (34.8)	29.7 (33.5)	33.3 (34.8)
Monogamous family	12.8 (24.5)	56.3 (51.8)	55.2 (60.1)	59.2 (58.9)
Monogamous extended family	17.0 (7.8)	1.2 2.4)	4.8 (0.6)	1.1
Polygamous extended family	9.5 (8.0)	7.2 (11.0)	10.3 (5.7)	6.3(6.4)
*N*	188 (163)	167 (164)	165 (158)	174 (141)
*Occupation of household head*				
Farmer/gardener	61.8 (63.0)	67.0 (90.6)	50.0 (46.0)	48.3 (71.2)
Trader/seller	3.9 (5.2)	0.6	1.8 (3.1)	15.3 (10.8)
Casual labourer	0.6	1.1 (1.3)	4.1 (1.8)	(0.7)
Civil servant	13.5 (20.1)	10.6 (4.4)	4.1 (3.1)	2.8 (1.4)
Pastoralist		0.6	0.6 (1.2)	26.7 (12.9)
Agropastoralist			1.8 (1.2)	
Gold mining		1.1 (0.6)	2.4 (5.5)	0.6
Fire wood sale		1.1	1.8 (0.6)	
Brewing		(3.1)	(4.3)	
Charcoal burning		15.1	31.8 (30.7)	1.1 (0.7)
Others	20.2 (11.7)	2.8	1.8 (2.5)	5.1 (2.2)
*N*	178 (154)	179 (159)	170 (163)	176 (139)
*Occupation of women*				
Farmer/gardener	94.8 (98.2)	70.4 (99.4)	53.8 (42.4)	91.4 (97.9)
Trader/seller	3.1 (1.2)	0.6	0.5	2.9 (1.4)
Casual labourer			2.7(4.8)	1.1(0.7
Civil servant	0.6			
Gold mining			(4.8)	
Fire wood sale		3.9	14.3 (10.3)	1.7
Brewing	1.6	10.6	7.1 (8.5)	
Charcoal burning	(0.6)	13.4 (0.6)	21.4 (29.1)	1.1
Others		1.1		1.7
*N*	192 (163)	179 (164)	182(165)	175 (141)
*Education level*				
No formal education	25.8 (27.2)	62.8 (66.3)	91.5 (89.6)	95.7 (94.3)
Primary education	55.8 (54.5)	36.6 (32.6)	8.5 (9.3)	4.3 (5.7)
Secondary education	17.8 (17.3)	0.6 (1.1)	(1.1)	
Tertiary/vocational training	0.6			
*N*	192 (163)	179 (164)	182(165)	175 (141)

The values in brackets are data for planting season; *N* is the sample size

In general, intake of energy and nutrients (Table 3) was higher during the planting season than in the harvesting season, except for the intake of calcium in Amudat district. In Abim district, intake of iron and zinc was significantly higher (*p* < 0.05), in the planting season than in the harvesting season by 5.8 mg/day and 1.4 mg/day. For Kaabong district, intake was significantly higher in planting season compared to harvesting season (*p* < 0.05) by 1009.6 kcal/day, 14.7 g/day, 93.7 g/day, 392 mg/day, 3.9 mg/day, 1.5 mg/day and 189.3 µg/day for energy, proteins, carbohydrates, calcium, iron, zinc and folate, respectively.

**Table 3:** Seasonal variability in energy and nutrient intake among women segregated by district

District	Season	Energy and nutrients													
		Energy (Kcal/d)		Proteins (g/d)		Carbohydrates (g/d)		Calcium (mg/d)		Iron (mg/d)		Zinc (mg/d)		Folate (dfe) (µg/d)	
		Mean	SD	Mean	SD	Mean	SD	Mean	SD	Mean	SD	Mean	SD	Mean	SD
Abim (*N* = 164)	A	1691.8	1098.3	51.5	33.7	266.9	154.7	503.5	535.6	16.6	12.1	8.1	5.5	274.6	186.0
	B	1813.8	1017.7	57.8	34.5	288.7	161.5	688.4	646.0	22.4	15.9	9.5	6.5	276.7	156.4
	*p* < *0.05*	0.211	0.08	0.138	0.05	0.000	0.026	0.900							
Kaabong (*N* = 165)	A	1398.8	638.0	44.6	22.3	276.8	125.7	313.9	431.4	13.8	6.6	7.3	3.8	120.9	77.9
	B	2408.4	1044.5	59.3	27.3	370.5	156.9	705.9	505.1	17.7	9.4	8.8	4.6	310.2	163.9
	*p* < *0.05*	0.000	0.000	0.000	0.000	0.000	0.001	0.000							
Moroto (*N* = 167)	A	1681.0	885.5	47.7	25.1	279.7	128.1	441.2	397.7	15.0	8.5	6.9	3.9	181.4	125.8
	B	2052.7	1002.8	54.7	27.2	336.9	166.9	438.8	327.6	16.7	8.9	7.8	4.2	252.0	151.7
	*p* < *0.05*	0.0	0.007	0.000	0.95	0.051	0.018	0.000							
Amudat (*N* = 142)	A	1898.3	708.7	54.8	23.7	323.5	127.8	1074.4	659.6	9.9	4.1	10.1	4.2	137.4	51.4
	B	2150.1	764.8	55.7	23.5	394.4	138.5	932.5	560.2	13.3	5.2	11.2	4.5	184.9	68.1
	*p* < *0.05*	0.003	0.714	0.000	0.042	0.000	0.026	0.000							

Seasons: A = harvesting season (October–December, 2014); B = planting season (April–June, 2015); *N* is the sample size

Similarly, in Moroto district, intake of energy, proteins, carbohydrates, zinc and folate was significantly higher (*p* < 0.05), in planting season than in harvesting season by 371.7 kcal/day, 7.0 g/day, 57.2 g/day, 0.9 mg/day and 70.6 µg/day. On the other hand, in Amudat district, energy, carbohydrates, iron, zinc and folate intake were significantly higher (*p* < 0.05) in planting season than harvesting season by 251.8 kcal/day, 70.9 g/day, 3.4 mg/day, 1.1 mg/day and 47.5 µg/day, respectively, while calcium intake was significantly higher (*p* < 0.05) in harvesting season than in planting season by 141.9 mg/day. Further, the result (Table 4) generally indicates significant variability in energy and nutrient intake among the districts irrespective of season.

**Table 4:** Variability in energy and nutrient intake among women disaggregated by district and season

Energy and nutrient intake	Season	District					
		Abim^a^ (*N* = 192(164))	Kaabong^b^ (*N* = 188(165))	Moroto^c^ (*N* = 184(165))	Amudat^d^ (*N* = 185 (142))	F pr. < 0.05	LSD (5%)
Energy (kcal)	1	1685.0 ± 1071^ab^	1400.0 ± 620.6^bad^	1714.0 ± 863.5^cb^	1851.0 ± 734.1^db^	< 0.001	169.8
	2	1814.0 ± 1018.0^abd^	2408.0 ± 1044.0^bac^	2078.0 ± 982.8^cab^	2150.0 ± 764.8^da^	< 0.001	195.0
Protein (g)	1	51.1 ± 32.5^ab^	44.8 ± 21.5^bad^	48.7 ± 24.9	53.1 ± 26^db^	0.017	5.4
	2	57.8 ± 34.5	59.3 ± 27.3	55.4 ± 26.7	55.7 ± 23.5	0.503	5.7
Carbohydrate (g)	1	267.0 ± 150.4^ad^	276.5 ± 124.6^bd^	286.9 ± 126.1^ cd^	316.4 ± 130.7^dabc^	0.003	26.9
	2	288.7 ± 161.5^abd^	370.5 ± 156.9^ba^	341.0 ± 163.7^cad^	394.3 ± 138.5^dac^	< 0.001	31.5
Fibre (g)	1	28.8 ± 17.0^abd^	24.9 ± 12.1^ba^	23.6 ± 12.2^ca^	24.9 ± 11.5^da^	< 0.001	2.7
	2	29.1 ± 18.1	26.6 ± 14.9^bd^	28.2 ± 15.0^ cd^	32.3 ± 13.3^dbc^	0.004	3.1
Calcium (mg)	1	476.8 ± 509.3^abd^	311.7 ± 414.4^bac^	449.4 ± 392.2^cbd^	1018.0 ± 702.1^dabc^	< 0.01	104.8
	2	688.4 ± 646.0^abcd^	705.9 ± 505.1^bacd^	444.1 ± 325.9.^cabd^	932.5 ± 560.2^dabc^	< 0.001	105.3
Iron (mg)	1	16.1 ± 11.6^abd^	13.8 ± 6.6^bad^	15.5 ± 8.5^ cd^	9.8 ± 4.4^dabc^	< 0.001	1.7
	2	22.4 ± 15.9^abcd^	17.7 ± 9.4^bad^	16.9 ± 8.7cad	13.3 ± 5.2^dabc^	< 0.001	2.2
Zinc (mg)	1	8.0 ± 5.3^acd^	7.2 ± 3.7^bd^	7.1 ± 3.9^cad^	10.0 ± 4.8^dabc^	< 0.001	0.9
	2	9.5 ± 6.5^acd^	8.8 ± 4.6^bd^	7.9 ± 4.1^cad^	11.2 ± 4.5^dabc^	< 0.001	1.0
Folate (dfe) (µg)	1	274.8 ± 191.1^abcd^	120.3 ± 79.7^bac^	185.9 ± 125.6^cabd^	134.7 ± 54.8^dac^	< 0.001	25.2
	2	276.6 ± 156.4^abd^	310.2 ± 163.9^bacd^	255.1 ± 150.0^cbd^	184.9 ± 68.1^dabc^	< 0.001	28.7

1 = harvesting season; 2 = planting season. a—Abim District, b—Kaabong District, c—Moroto District, d—Amudat District. Significant differences among districts are indicated as superscripts on intake levels of different nutrient

In general, irrespective of the district, physiological status and season, household consumption of plant-based foods was far higher than intake of animal-based foods (Table 5). Among the plant-based foods, intake was dominated by starchy stables, dark green leafy vegetables and legumes, nuts and seeds. In the case of animal-based foods, consumption of milk was highest, followed by other meat and fish, eggs and organ meat. The highest proportion of households that consumed milk was in Amudat, followed by Moroto, Kaabong and Abim.

**Table 5:** Proportion of participants who consumed various food groups segregated by physiological status, season and district

District	Physiological status	Seasons	*N*	Proportion (%) of women of childbearing age that consumed various food groups									
				Starchy staples	Dark green leafy vegetables	Other vitamin A-rich fruits &vegetables	Other fruits &vegetables	Organ meat	Meat and fish	Eggs	Legumes, nuts and seeds	Milk and milk products	Alcoholic beverages
Abim	Lactating	A	65	76.8	73.8	18.7	67.7	1.5	14.6	12.5	87.7	9.2	60
		B	95	97.9	68.4	7.5	64.5	0	13.2	4.2	89.5	3.15	68
	Pregnant	A	65	84.4	85.2	27.1	63.9	0	23.4	14.5	96.7	17.5	46.1
		B	20	95	90	25	85	0	7.5	0	95	5	60
	Non-pregnant	A	62	86.9	77	32.4	65.6	6.6	20.9	10	95.1	16.1	63.3
		B	49	93.9	75	20.4	67.3	2	9.15	2	85.7	2	57.1
Kaabong	Lactating	A	64	100	89.1	32.8	83.3	1.6	31.3	15.6	96.9	36.2	92.2
		B	92	100	91.3	16.3	46.7	0	20.7	4.3	56.5	54.3	91.3
	Pregnant	A	63	95.2	87.3	22.3	62.3	1.6	23.3	12.7	65.1	30.95	85.7
		B	19	100	100	5.3	42.1	0	5.3	0	42.1	36.8	84.2
	Non-pregnant	A	63	98	85.7	37.3	82.5	4.8	25.4	4.8	81	26.2	74.6
		B	54	100	83.3	11.1	44.4	0	14.8	7.4	70.4	46.3	92.6
Moroto	Lactating	A	68	100	92.6	22.1	36.8	1.5	14.7	4.4	60.3	58.8	69.1
		B	104	99	91.3	17.3	55.8	1	8.7	3.8	57.7	40.2	89.4
	Pregnant	A	63	100	84.1	12.7	55.6	4.8	12.8	4.8	65.1	50.8	68.3
		B	14	100	71.4	42.9	57.1	0	25	7.1	71.4	50	92.9
	Non-pregnant	A	53	98.2	78.2	7.3	44.4	1.8	10.9	0	61.8	50.9	65.5
		B	47	100	91.5	34	59.6	0	14.9	0	76.6	38.3	87.2
Amudat	Lactating	A	70	97.1	76.8	8.7	47.8	1.4	20.3	5.8	21.7	76.6	4.2
		B	90	100	82.2	2.2	22.2	0	36.7	15.6	38.9	84.4	1.1
	Pregnant	A	62	98.4	69.4	11.3	50	3.2	27.4	14.5	27.4	83.9	4.8
		B	20	100	95	0	40	0	30	10	35	80	0
	Non-pregnant	A	53	98.1	69.8	9.4	43.4	0	22.6	13.2	30.2	84.9	1.9
		B	31	100	80.6	6.5	12.9	0	38.7	22.6	35.5	80.6	0

A = harvesting season; B = planting season; *N* is the sample size; Alcohol is not a conventional food group; it is included among the food groups because of its high level of consumption in the sub-region

**Fig. 1:**
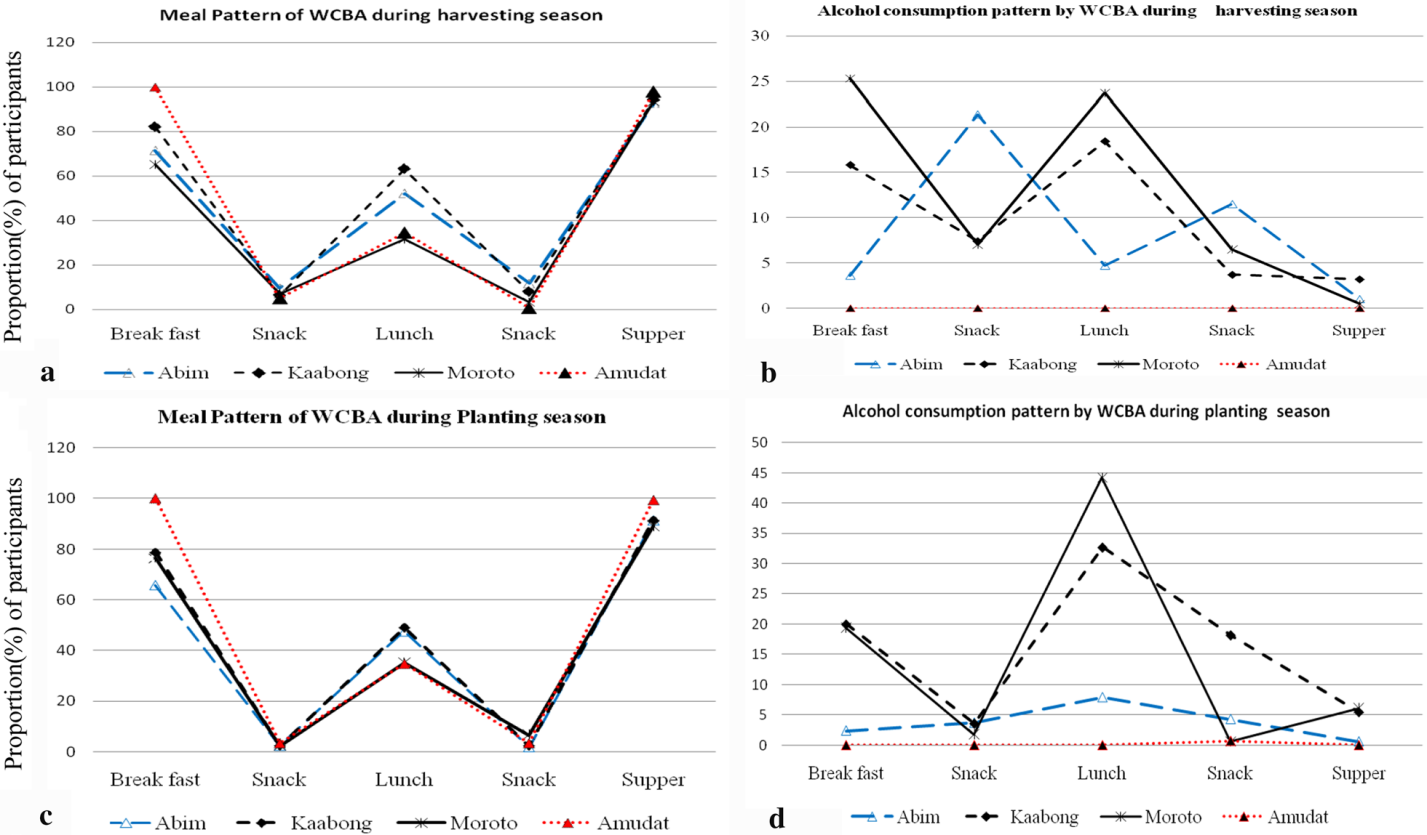
Meal patterns and alcohol consumption patterns among WCBA according to season (harvesting and planting seasons) and district

The meal times had a similar pattern (Fig. 1a and c) among all the respondents with the majority taking breakfast and supper while lunch was consumed by a relatively small proportion of women (35–65%). Alcohol consumption in Amudat was relatively low compared to the situation in Kaabong and Moroto (Fig. 1b and d). Locally brewed alcohol was consumed at breakfast, lunch and supper and snack time, either as a full meal or as a supplementary to full meal.

According to Table 6, the proportion of pregnant women that had inadequate intake of energy, protein, carbohydrate, calcium, iron and zinc was generally higher in the harvesting season than in the planting season. With exception of carbohydrates, most of the pregnant women did not meet the RDA for energy and nutrients. Lactating mothers who did not meet RDAs for energy and nutrients accounted for 86–100% for folate, 54–97% for calcium, 66–88% for zinc, 66–89% for protein, 58–84% for energy, 4–37% carbohydrates and 15–30% for iron in decreasing order of magnitude. Similarly, non-pregnant women who did not meet the RDAs was the highest for folate (74–100%) followed by calcium (47–96%), iron (49–96%) zinc (25–75%), energy (29–73%), protein (37–59%) and carbohydrate (0–18%) in decreasing order of magnitude.

**Table 6:** Levels of inadequacy of energy and nutrient intake among WCBA segregated by physiological status, district and season

Physiological status	Districts	Season	*N*	Proportion (%) that had adequate intake of energy and nutrient						
				Energy	Protein	Carbohydrates	Calcium	Iron	Zinc	Folate
Pregnant women	Abim	A	65	84.6	87.7	38.5	92.3	93.8	92.3	95.4
		B	20	75	75	15	85	75	75	100
	Kaabong	A	63	90.5	84.1	19	92.1	95.2	88.9	100
		B	19	36.8	68.4	0	68.4	78.9	63.2	100
	Moroto	A	63	69.8	79.4	25.4	92.1	88.9	84.1	100
		B	14	42.9	64.3	28.6	100	85.7	71.4	92.9
	Amudat	A	62	64.5	85.5	11.3	45.2	100	67.7	100
		B	20	35	70	0	50	95	50	100
Lactating mothers	Abim	A	65	67.7	72.3	36.9	86.2	18.5	75.4	86.2
		B	95	73.7	72.6	36.8	82.1	16.8	73.7	89.5
	Kaabong	A	64	84.4	89.1	25	96.9	18.7	87.5	98.4
		B	92	41.3	66.3	14.1	78.3	15.2	71.7	88
	Moroto	A	68	63.2	76.5	20.6	85.3	17.6	77.9	95.6
		B	104	57.7	74	15.4	92.3	18.3	81.7	96.2
	Amudat	A	70	58.6	72.9	21.4	54.3	30	65.7	100
		B	90	57.8	80	4.4	62.2	20	66.7	100
Non-pregnant women	Abim	A	62	54.8	53.2	17.7	87.1	64.5	56.5	74.2
		B	49	40.8	36.7	10.2	73.5	49	42.9	73.5
	Kaabong	A	63	73	58.7	14.3	93.7	74.6	65.1	98.4
		B	54	27.8	37	3.7	75.9	68.5	51.9	79.6
	Moroto	A	55	65.5	60	9.1	92.7	69.1	74.5	94.5
		B	47	40.4	48.9	8.5	95.7	70.2	68.1	87.2
	Amudat	A	53	50.9	43.4	7.5	47.2	96.2	41.5	100
		B	31	29	41.9	0	54.8	87.1	25.8	100

A = harvesting season; B = planting season; *N* is the sample size; RDAs for pregnant women: energy, 2100 (Kcal/d); protein, 71 (g/d); carbohydrate, 175 (g/d); calcium, 1000 (mg/d); Iron, 27 (mg/d); zinc, 11 (mg/d); folate, 600 (µg/d); RDAs for lactating mothers: energy, 2160 (Kcal/d); protein, 71 (g/d); carbohydrate, 210 (g/d); calcium, 1000 (mg/d); iron, 9 (mg/d); Zinc, 12 (mg/d); folate, 500 (µg/d); RDAs for non-pregnant women: energy, 1710 (Kcal/d); protein, 46 (g/d); carbohydrate, 130 (g/d); calcium, 1000 (mg/d); iron, 18 (mg/d); zinc, 8 (mg/d); folate, 400 (µg/d); ^1^values for fibre indicates proportions of WCBA with intakes above the RDA

The socio-economic and demographic predictors of inadequate intake of energy and nutrients vary between seasons and among nutrients (Table 7). For energy in the harvesting season, being a lactating or breastfeeding mother increased inadequacy of intake (*β*: 0.152; *p* < 0.05). Inadequacy in intake of calcium significantly reduced due to an increase age (*β*: − 0.099) and occupation of the household head (*β*: − 0.111) during harvesting season while inadequacy in the intake of the same nutrient increased (*p* < 0.05) during harvesting (*β*: 0.100) and planting season (*β*: 0.199) due to the occupation of the spouse. Inadequacy in the intake of zinc increased (*p* < 0.05) due to occupation of the spouse (*β*: 0.126). Inadequacy in the intake of folate increased (*p* < 0.05) due to an increase in age (*β*: 0.090) during planting season. As far as vitamin A is concerned, inadequacy of intake increased (*p* < 0.05) due to being married (*β*: 0.127), occupation of the spouse (*β*: 0.135) and the number of women married in the household (*β*: 0.132) during harvesting season. For the same nutrient during harvesting season inadequacy of intake reduced (*p* < 0.05) due to an increase in age (*β*: − 0.126), occupation of the household head (*β*: − 0.151), education of the household head (*β*: − 0.229). Further, education of the spouse reduced inadequacy of intake of vitamin A during both harvesting (*β*: − 0.082) and planting seasons (*β*: − 0.114).

**Table 7:** Linear regression analysis of socio-demographic predictors of inadequate intake of energy and nutrients among WCBA segregated by season

Socio-demographic predictors	Season	Inadequacy of energy and nutrient intake^1^						
		Energy	Proteins	Calcium	Iron	Zinc	Folate (dfe)	Vitamin A (rae)
Pregnancy (*X*_1_)	A	− 0.059	0.036	0.004	0.029	0.033	0.027	− 0.021
	B	− 0.008	− 0.008	− 0.008	− 0.007	0.018	0.012	− 0.055
Lactating//breastfeeding (*X*_2_)	A	0.152*	− 0.003	0.007	0.022	0.001	− 0.007	0.036
	B	0.016	0.015	0.019	− 0.007	0.005	0.015	− 0.054
Age (*X*_3_)	A	− 0.011	− 0.040	− 0.099*	− 0.040	− 0.045	− 0.024	− 0.126*
	B	0.043	− 0.022	0.024	0.001	− 0.049	0.090*	− 0.007
Polygamous (*X*_4_)	A	0.024	0.036	0.026	0.036	0.036	0.037	0.013
	B	0.057	0.096	0.018	0.063	0.073	0.067	0.055
Marital status (married) (*X*_5_)	A	− 0.006	0.006	0.017	− 0.009	0.009	− 0.008	0.127*
	B	− 0.033	− 0.009	0.018	− 0.070	0.039	− 0.063	0.041
Occupation of household head (*X*_6_)	A	− 0.049	− 0.057	− 0.111*	− 0.028	− 0.059	− 0.055	− 0.151*
	B	− 0.019	− 0.037	− 0.062	− 0.034	− 0.086	0.034	− 0.070
Occupation of the spouse (woman) (*X*_7_)	A	− 0.014	0.040	0.100*	0.015	0.045	0.044	0.135*
	B	− 0.019	0.017	0.199*	− 0.016	0.126*	− 0.089	− 0.087
Education level of household head (*X*_8_)	A	− 0.042	0.043	− 0.066	0.071	0.035	0.063	− 0.229*
	B	− 0.043	0.002	− 0.002	0.069	− 0.078	0.109	0.063
Education level of spouse (woman) (*X*_9_)	A	− 0.038	0.005	0.002	0.007	0.006	0.012	− 0.082*
	B	− 0.052	0.019	− 0.065	0.091	0.026	0.050	− 0.114*
Number of women married (*X*_10_)	A	− 0.028	0.005	0.056	− 0.002	0.007	− 0.009	0.132*
	B	0.057	0.080	0.049	0.001	0.078	0.044	− 0.021

A and B denote harvesting and planting seasons, respectively; *The demographic factor is a predictor at 5% significance level. ^1^Regression coeffiients (*β*) of inadequate intake are presented for energt and nutrients (proteins, calcium, iron, zinc, folate and vitamin A)

Regarding nutrition status overall across districts and physiological status, the prevalence of malnutrition was higher in harvesting season than in planting season. Abim had the lowest prevalence of malnutrition across all the seasons and physiological status (Table 8). Among pregnant women, the prevalence of moderate malnutrition ranged from 9.2% in Abim district to 24.2% in Amudat district during harvesting season while during planting season, the prevalene ranged from 4.9% in Abim district to 16.4% in Moroto district. Among lactating mothers, the highest prevalence of malnutrition during harvesting occurred in Moroto (25.4%) followed by Kaabong (23.8%), Amudat (17.3%) and Abim (7.7%) while during planting season, the prevalence of malnutrition was highest in Amudat and Moroto (16.4%) followed by Kaabong (10%) and Abim (3.6%) in decreasing order of magnitude. Prevalence of malnutrition among non-pregnant women during harvesting season was highest in Kaabong (27.2%) followed by Moroto (17.6%), Amudat (15.1%) and Abim (3.2%). During planting season, prevalence of malnutrition had a similar pattern accounting for 15.7% among non-pregnant women in Kaabong, 13.3% in Moroto, 12.2% in Amudat and 0% in Abim (Table 8). The output from correlation between MUAC and nutrient intake (Table 9) indicate that during harvesting season, MUAC is positively correlated with protein in Amudat and Kaabong, zinc intake (*p* < 0.05). On the other hand, there was a significant negative correlation (*p* < 0.05) of MUAC with intake of calcium in Amudat district during planting season.

**Table 8:** Prevalence of undernutrition among women of different physiological status segregated by district and season

Physiological status	District/ seasons	*n*		Prevalence (%) of under-nutrition	
		1	2	1	2
Pregnant	Abim	65	56	9.2	4.9
	Kaabong	62	55	22.6	12.7
	Moroto	61	55	22.9	16.4
	Amudat	62	47	24.2	12.8
Lactating	Abim	65	55	7.7	3.6
	Kaabong	63	60	23.8	10
	Moroto	63	61	25.4	16.4
	Amudat	69	56	17.3	16.4
Non-pregnant	Abim	62	51	3.2	0
	Kaabong	62	54	27.2	15.7
	Moroto	51	45	17.6	13.3
	Amudat	53	40	15.1	12.2

1 = Harvesting Season; 2 = Planting Season; *n* is the sample size

Prevalence of undernutrition was calculated based on MUAC cut-off point of < 23.0 cm [49]

**Table 9:** Correlation between MUAC and nutrient intake segregated by district and season

Energy and nutrients	Abim		Amudat		Kaabong		Moroto	
	1 (*n* = 192)	2 (*n* = 162)	1 (*n* = 183)	2 (*n* = 143	1 (*n* = 185)	2 (*n* = 169)	1 (*n* = 176)	2 (*n* = 160)
Energy	0.030	0.013	0.142	0.027	0.093	0.010	0.069	0.030
Proteins	− 0.010	− 0.014	0.149^*^	0.001	0.157^*^	− 0.012	− 0.009	0.015
Carbohydrates	0.011	0.012	0.107	− 0.045	0.061	0.015	0.010	0.045
Fibre	− 0.019	− 0.052	0.098	0.024	0.070	0.021	− 0.001	0.045
Calcium	− 0.007	0.020	0.097	− 0.089	0.122	− 0.050	− 0.029	− 0.002
Iron	− 0.013	− 0.039	0.138	− 0.165^*^	0.093	− 0.097	− 0.090	− 0.029
Zinc	0.007	− 0.045	0.140	− 0.111	0.146^*^	− 0.042	0.017	0.036
Folate	0.067	− 0.033	0.132	0.064	0.025	− 0.024	0.119	0.053

1: harvesting season; 2: planting season; Values are correlation coefficients; *Correlation coefficients significant at 5% level

## Discussion

Results presented in Table 2 suggest low socio-economic status characterized by subsistence farming, low education levels, low occupation levels, polygamous family settings, conditions that predispose WCBA to poor dietary intake and, consequently, nutrition status. Such low socio-economic status coupled with complex demographic factors can present a critical bottle neck to the success of nutrition interventions in Karamoja sub-region. From a policy perspective, efforts that lead to improvement of such socio-demographic and economic conditions are likely to have a positive nutrition outcome among WCBA in Karamoja sub-region.

The average intake of energy and nutrients fluctuates among the districts varies between the seasons was generally high in the second season (Table 4). The average of energy and nutrients an important fact about individual level intakes as depicted by large standard deviations (Table 3). The differences in the average intakes of energy and nutrient intake can be attributed to socio-economic and demographic factors that drive livelihood strategies at different seasons (Table 2). The result (Table 3) indicates that generally, the intake of energy and nutrients was significantly higher during planting season than harvesting season. This is contrary to what is expected during harvesting season. However, according to FEWWSNET 2019 report [50], food security begins to improve in Karamoja as the harvest begins and sorghum prices decline. One of the probable reasons is based on the observation during data collection that harvesting of most of staple foods such as sorghum span up to February and food was still adequate at the households during the second phase of data collection (planting season). The high proportion of pregnant women failing to meet RDAs for folate, iron and zinc (Table 6) can be attributed to their high micronutrient requirements in this physiological category than women in other physiological status [47] and consumption of foods poor in micronutrients (Table 5). This suggests that women were generally nutritionally deficient before they conceived and experienced even higher deficiencies during pregnancy and lactation owing to their failure to meet the increased energy and nutrient requirements. Nutrient intake in the prenatal period is one of the most important determinants of foetal growth and development and supports maternal health [51–53]. Such high demand for nutrients and energy makes pregnant women more vulnerable to malnutrition resulting in both short-term effects such as miscarriages, low birthweight, low maternal weight, frequent disease episodes and long-term effects such as non-communicable diseases (obesity, type 2 diabetes, hypertension and cardiovascular diseases) in later life [54]. The result therefore implies that pregnant women in Karamoja sub-region are predisposed to inadequate dietary intake which can impact negatively foetal growth and later on the health and nutrition status of the children.

As shown in Table 6, most of the non-pregnant/non-lactating mothers did not meet RDAs for micronutrients in both seasons. This implies that from nutritional point of view, most mothers conceive when they are undernourished. This is consistent with the results of the current study (Table 8) where except Abim district; there was a high prevalence of malnutrition among the non-pregnant women. The nutritional demand by lactating women is also considerably greater than when pregnant, while diet can affect the synthesis, composition and secretion of milk [55]. Mothers who are well nourished during pregnancy will have adequate fat and other nutrient reserves to fall back on when lactating [56]. It is apparent that women in Karamoja sub-region do not consume adequate nutrients to support their health and the health of children that nutritionally depend on them. Compromised nutrient intake during the critical period immediately before and following conception can adversely influence pregnancy outcome, increases risk of birth defects, can alter nutrient composition of breast milk, condition infants and children to altered growth trajectories and predisposes them to chronic diseases in later stage of life [57]. While the effects of pre-pregnancy dietary intake are not well characterized, improvements prior to pregnancy may decrease the risk of poor maternal and foetal outcomes [38].

As shown in Table 5, plant-based foods constitute a major part of the household diet in Karamoja sub-region and consumption varies from season to season. This consistent with the literature that most diets in a SSA are plant based [58]. The positive contributions of such plant-based foods to micronutrients and other bioactive substances [59] are of limited by antinutritional factors (e.g.; phytates, polyphenols, oxalates, tannins) [60, 61] affecting already heightened nutrient needs of WCBA in Karamoja sub-region.

Regarding seasonality, a similar study [62] that examined dietary diversity and the nutritional status of women in rural Burkina Faso reported that dietary diversity scores (DDSs) were sensitive to seasonal variations. Their findings are in agreement with the current study (Table 3) which showed variation in energy and nutrient intake and consumption of food groups between the harvesting and the planting seasons. Whereas it is generally recommended that pregnant women should consume three meals and two or more snacks per day to reduce the risk of preterm delivery [63], this was not the case for WCBA in Karamoja sub-region (Fig. 1a and c) and was complicated by consumption of alcohol by WCBA (Fig. 1b and d). Prolonged periods of time without food may induce physiological stress during pregnancy [63] while alcohol consumption increases the risk of physical and mental damage to their babies referred to foetal alcohol syndrome [64] adversely affect micronutrient absorption and availability during that physiological state [37]. During period of breastfeeding, alcohol consumption may place newborns at an additional disadvantage as teratogenic effects of alcohol are increased when micronutrients such as iron, zinc and choline are deficient [37]. The habitual intake of alcohol in Karamoja by WCBA observed in the current study suggests that children in Karamoja sub-region are more likely to experience mental and behavioural challenges such as deficits in cognitive functioning (such as general intellectual functioning, learning of new verbal information and performance on visual-spatial tasks) and fine- and gross-motor performance [65]. It can therefore be asserted that WCBA in Karamoja sub-region is at high risk of malnutrition and poor health during the season of inadequate food intake (harvesting season) and that these risks have important implications for the well-being and development of children in the community.

Linear regression on the 10 independent variables reported in the current study (Table 7) significantly predicted inadequate intake of energy and selected mircronutrients (calcium, zinc, folate and vitamin A) among WCBA depending on the seasons. It is not surprising to note that being a lactating mother predicted limitations in the intake of energy. This is because lactation/breastfeeding is often associated with increased energy need, requiring the mothers to increase dietary energy intake above that recommended for normal adult women to support breast milk production and prevent maternal malnutrition [66]. Unfortunately, in Karamoja sub-region, the positive coefficients for inadequate energy intake imply that energy among lactating mothers was low. This situation has serious ramifications for nutrition and health well-being of these mothers and children in Karamoja sub-region. This is consistent with previous studies that inadequate energy intake leads to poor health and nutrition outcomes [67] and possibly increased risk of mortality [68, 69].

The current study (Table 7) also indicates that a unit increase in maternal age and occupation of the household head was associated with a decrease in inadequate intake of calcium but increased inadequacy of intake of folate among WCBA. In addition, increase in education level of both the spouse and the household head was associated with a decrease in inadequacy of intake of vitamin A. A reduction in inadequacy of calcium intake with age suggests that pregnant and lactating women are less likely to experience calcium deficiency as they grow old. However, this is a subject matter proposed for further investigation. The findings of the current regarding education level is in agreement with a study involving 530 pregnant women at Ile-Ife in Nigeria [68], which indicated that overall, there was a significant relationship between the level of education and knowledge and dietary intake. Education plays a key role in determining maternal under-nutrition [70]. According to Serbesa et al.[71], education status of pregnant women and lactating mothers affected nutrition in the sense that being educated was associated with a higher income, ability to make better decisions for her nutrition and that of the child. The authors further argued that educated pregnant women and lactating mothers were more careful about what they ate than the uneducated counterparts. A similar study [72] also affirmed that antenatal mothers with good education background had higher nutritional knowledge and enhanced understanding of information disseminated through mass media. However, the results of the current study (Table 2) indicate high proportion of women who never attained any formal education, thus suggesting limited ability of WCBA in Karamoja sub-region to comprehend nutrition information provided. Considering the significance of nutrition information in fostering good nutrition behaviours [73, 74], adult education could be implemented to improve education status of WCBA in Karamoja sub-region. Occupation of women limited the intake of calcium, zinc and vitamin A depending on season and nutrient type (Table 7). The observed disparity in the influence of occupation on intake of nutrients in the current study might have been confounded by such dichotomy. This is consistent with the long-held view that results of regression models should be interpreted with care [75], taking in to account the dichotomy that exists in terms of types and quantity of foods available for consumption between planting and harvesting seasons by reporting the adjusted coefficients [76]. The influence of occupation on maternal nutrition was argued in previous studies that women who are employed outside the home for long duration have less leisure time because of work pressure, and hence they cannot take proper care of their health and frequently neglect dietary intake [77]. For the case of Karamoja, due to low education attainment, most of the inhabitants are not formally employed. Inadequacy in the intake levels of nutrient due to occupation could arise from the fact that most of the women engage in subsistence agriculture (Table 2) where they spend long hours in the garden without paying attention to their dietary intake.

Being married is generally believed to be of benefit for maternal nutrition. It has been argued that being married enhances family income and wealth, provide social support and other noneconomic resources that help individuals withstand periods of economic uncertainty or stress, though the benefits of marital support may be smaller for women than men [78]. However, the results of the current study indicate that being married was associated with increased inadequacy of vitamin A intake among WCBA. Thus, illustrating from a nutrition point of view and based on the results, there is limited efficacy of marital support in fostering nutritional well-being of WCBA in a food insecure location such as the Karamoja sub-region. The significance of marital status in nutrition well-being of WCBA seems to be dependent on the nature of marriage type (monogamy and polygamy). This is because polygamy tends to have a negative effect on energy and nutrients, confirmed by the result that an increase in the number of women married in a household increased inadequacy of intake of vitamin A (Table 7). This finding implies that marrying more than one woman in a food insecure environment as is the case in Karamoja sub-region predisposes WCBA and other vulnerable members of the household such as children to more food and nutrition insecurity, and malnutrition. Considering the positive impact of adequate nutrition on health well-being of WCBA, children [79] and human-factor-related development [80–83], community dialogue could be attempted to discourage polygamy if food and nutrition security situation in the sub-region persists.

Lastly, it is widely recognized that dietary intake affects nutrition status of individuals. For example a positive linear relationship was established between dietary intake and nutrition status of pregnant women [84]. The results of the current study (Table 8) indicate that with the exception of Abim district, women (10–25.4%) regardless of physiological status in both seasons in Kaabong, Moroto and Amudat were malnourished as assessed by MUAC. It can therefore be asserted that WCBA in Karamoja sub-region are at high risk of malnutrition and poor health during the season of inadequate food intake (harvesting season), and that these risks have important implications for the well-being and development of children in the community. The positive relationship between MUAC, and protein and zinc (Table 9) is consistent with the findings of previous studies [85] that reported positive association of MUAC with markers of protein nutrition and micronutrient status. From this and the result of the current study, it can therefore be deduced that increased protein intake can lead to increase MUAC. However, the negative correlation between MUAC and intake in Amudat (Table 9) could be a result of a combination of factors low iron intake (Table 3) and inhibitory effect of high intake of calcium on iron absorption [86]. Low-dietary iron intake results in anaemia [87, 88] and consequently weight gain due to fatigue and limited physical activity [89].

Potential limitations to data collection process were that the data could have been affected by recall bias due to the 24-h recall period and that the estimates for nutrients did not take into consideration their bioavailability. In addition, data were collected once in each season which could have influenced the reliability of the results. Hence, there could have been over estimates or under estimates as a result of measuring the portion sizes. However, because the data were obtained from a fairly large sample size, these findings provide important information regarding the intake levels of energy and nutrients among WCBA. More so, the current study did not focus on energy expenditure among WCBA. We propose that future studies consider a detailed investigation of energy expenditure by WCBA.

## Conclusion

This study demonstrated that energy and nutrient intake among WCBA differed between the harvesting and planting seasons, with high levels of variability within each district surveyed. WCBA had inadequate energy and nutrient intake based on the recommended daily allowances. WCBA in largely consume plant-based diets, notably starchy staples and green vegetables, as well as legumes, nuts and seeds. WCBA in Karamoja sub-region, regardless of physiological status consume large amounts of locally produced alcohol, either as a meal or as a supplement to a meal. Lactation/breastfeeding, age of WCBA, number of women married, education level and occupation of the household head and spouse were the key socio-economic and demographic factors that influenced inadequate intake of energy and nutrients among WCBA depending on season.

On the basis of the findings of the current study, it is recommended that interventions to address maternal undernutrition reflect fine-grained seasonal differences within the sub-region if they are to be successful in achieving good nutrition outcomes for WCBA and their children.

Government and development agencies should provide nutrition education to both women and men to increase their knowledge on good feeding practices, nutritious foods, and balanced diet among others to reap the benefits including increased intake levels of nutrients, improved dietary diversity and improved nutrition status. This can be achieved through food and cooking demonstrations using locally available foods. Added to this, due to high levels of illiteracy among women in Karamoja sub-region, such interventions need to be thoughtfully and sensitively conceived and implemented using simple methods such as pictures, practical demonstrations, drama, music and dance, and community dialogue.

## Data Availability

The data sets used and/or analysed during the current study are available from the corresponding author on reasonable request.

## Abbreviations

ACF: Action Against Hunger
DHS: Demographic Health Survey
EC: European Commission
FANTA: Food and Nutrition Technical Assistance
FAO: Food and Agriculture Organization
FEWS Net: Famine Early Warning System Network
HDDS: Household Dietary Diversity Sore
IPC: Uganda IPC Technical Working Group
LMICs: Low- and Middle-Income Countries
NEJOGHA: Network of Climate Journalists of the Greater Horn of Africa
OPM: Office of the Prime Minister
RDA: Recommended daily allowance
SSA: Sub-Saharan Africa
SPSS: Statistical Package for Social Scientists
SUN: Scaling Up Nutrition
UBOS: Uganda Bureau of Statistics
UNAP: Uganda Nutrition Action Plan
UNCST: Uganda National Council for Science and Technology
UNDP: United Nations Development Programme
UNOCHA: United Nations Office for the Coordination of Humanitarian Affairs
UNSCN: United Nations System Standing Committee on Nutrition
USAID: United States Agency for International Development
WCBA: Women of Childbearing Age
